# Broma and Dietetic Cocoa

**Published:** 1853-07

**Authors:** 


### MATERIA MEDICA AND THERAPEUTICS.

*Broma and Dietetic Cocoa.*—Every body in New England, of course, is quite familiar with those two excellent articles of diet for invalids, broma and dietetic cocoa, manufactured by Walter Baker, of Dorchester, Mass. Some years since, the special consideration of medical practitioners was called to these preparations, as appropriate food for the sick, in the various conditions of debility and prostration to which they are at times reduced, leaving the digestive apparatus too feeble to appropriate any but the most delicate nutriment. Medical gentlemen of eminence in this city were delighted with Mr. Baker's broma; and from that period to this, its good character has been sustained. Another set of physicians have commenced business since that period, who may not have become familiar with the article; and we therefore refer again to the subject, for the purpose of reminding both our young medical friends at home and those at a distance, that they will derive important advantages from the use of these admirable kinds of food. Druggists in the interior would find their account in always keeping both on hand, with a view to meeting the prescriptions of medical attendants. From our own personral experience of the value of broma particularly, we can speak decidedly in its favor. A dietetic course is not unfrequently quite as necessary as strict medication; and in recovering from a low state, it is one of the perplexities of a general practitioner's life, to determine what may or may not be safely adopted as a regimen.—*Boston Med. and Surg. Journ.*

